# Participatory praxis as an imperative for health-related stigma research

**DOI:** 10.1186/s12916-019-1263-3

**Published:** 2019-02-15

**Authors:** Laurel Sprague, Rima Afifi, George Ayala, Musah Lumumba El-nasoor

**Affiliations:** 1 Joint United Nations Programme on HIV/AIDS (UNAIDS), Geneva, Switzerland; 2 College of Public Health, University of Iowa, Iowa City, Iowa USA; 3 MPact Global Action for Gay Men’s Health and Rights, Oakland, California USA; 4 Uganda Youth Coalition on Adolescent SRHR and HIV, Busia, Uganda

**Keywords:** HIV/AIDS, stigma, health, community engagement, participatory praxis, community-based participatory research

## Abstract

**Background:**

Participatory praxis is increasingly valued for the reliability, validity, and relevance of research results that it fosters. Participatory methods become an imperative in health-related stigma research, where the constitutive elements of stigma, healthcare settings, and research each operate on hierarchies that push those with less social power to the margins.

**Discussion:**

Particularly for people who are stigmatized, participatory methods balance the scales of equity by restructuring power relationships. As such, participatory praxis facilitates a research process that is responsive to community-identified priorities and creates community ownership of the research, catalyzing policy change at multiple levels and foregrounds, and addresses risks to communities from participating in research. Additionally, through upholding the agency and leadership of communities facing stigma, it can help to mitigate stigma’s harmful effects. Health-related stigma research can reduce the health inequities faced by stigmatized groups if funders and institutions require and reward community participation and if researchers commit to reflexive, participatory practices. A research agenda focused on participatory praxis in health-related stigma research could stimulate increased use of such methods.

**Conclusion:**

For community-engaged practice to become more than an ethical aspiration, structural changes in the funding, training, publishing, and tenure processes will be necessary.

## Background

Participatory praxis is increasingly valued for the reliability, validity, and relevance of research results that it fosters [1–3]. As a collection of research methods that document, acknowledge, and respect local knowledge, participatory praxis provides an approach to negotiating differences between researchers and community members such that the research perspective does not supersede community perspectives or subordinate the community in its intent or its outcomes [4]. These participatory methods take their starting point from the strengths and assets inherent in a community, rather than from a weakness and deficit perspective. Participatory praxis holds heightened importance for health-related stigma research, which focuses on the “*status loss and social rejection*” [5] that arise when people with, or associated with, specific health or social conditions are labeled as different and treated as undesirable, resulting in significant health inequities and disparities [5, 6]. In health-related stigma research, the constitutive elements of stigma, healthcare settings, and research each operate on hierarchies that push those with less social power to the margins, risking further marginalization in the name of knowledge production [7]. These hierarchies are magnified when researchers from high-income countries conduct research in low- and middle-income countries without engaging local researchers or local knowledge. However, health-related stigma research can have the opposite effect – reducing the marginalization and resulting health inequities faced by stigmatized groups – if researchers commit to reflexive, participatory practices and funders and institutions require and reward meaningful community participation.

This opinion piece argues that participatory praxis in health-related stigma research is an imperative. Particularly for people who are stigmatized, participatory praxis can balance the scales of equity by restructuring power relationships. This is because participatory praxis in health-related stigma research inherently strives to reveal insidious power structures, expose biases, and enrich understanding of community strengths and health needs. In so doing, it offers communities that are disenfranchised opportunities to exercise agency, leadership, and value to their communities. In the next sections, we highlight key aspects of participatory praxis relevant to persons who are stigmatized, suggest needed structural changes to bolster this approach, and propose a preliminary research agenda.

Our opinion piece approaches this topic from the perspective of researchers working with disenfranchised and stigmatized communities, and offers guidance from this reality. We acknowledge that much of the movement towards participatory praxis has arisen from the struggles and revolts of those communities themselves, and their agency and voice [8]. In what follows, we do not intend to minimize these contributions, rather to offer a critical analysis that intends to lift up those struggles, revolts, and voices as a precursor to research for social change.

## Discussion

### Continuum of participation

**Fig. 1:**
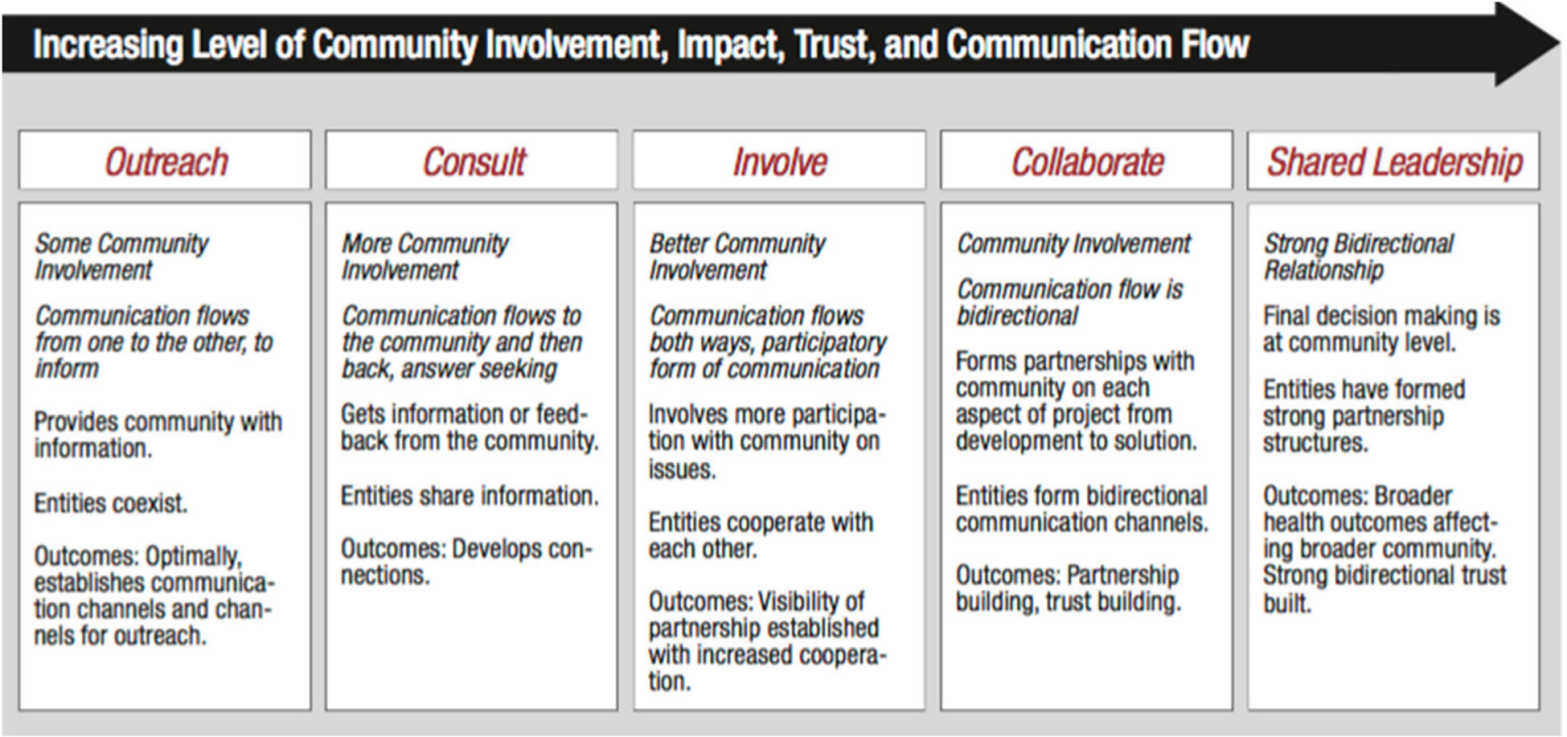
Continuum of community participation in research. Adapted from [9] with permission from the International Association for Public Participation

Community participation exists on a continuum, with one-way communication from researchers to communities at one end and activities that constitute participatory praxis through shared leadership at the other (Fig. 1). These activities include communities actively identifying questions, reviewing protocols to maximize participation and protection of vulnerable participants, implementing research methodologies, helping to interpret results within appropriate contexts, and applying results to influence decisions [4, 9, 10]. On this continuum, research with minimal engagement results in further objectification of stigmatized people, heightening risks for discrimination, humiliation, criminalization, and violence. By contrast, participatory methods result in enhanced agency, dignity, and wellbeing [2, 4].

Working within this continuum, there are different kinds of participatory praxis as well as different levels of engagement. In situations in which researchers are unable at a given point in time to reach shared leadership, a clear articulation of values, principles, and orientation are useful for conceptualizing how to ensure stigmatized communities are engaged in ways that provide a maximum amount of respect, agency, and dignity. Principles and orientations have been described extensively in community-based participatory research – one form of participatory praxis – and include commitment to co-learning, capacity-building, joint benefit, decreasing inequities, and social change [4, 11, 12]. We expand on these values below. We posit that – at every point in the research process (from development of research questions to dissemination of results) – realization of these principles, orientation, and values is possible and necessary, even if the goal of shared leadership is not achievable in the short term. Moreover, research projects that are initially characterized by the ownership and control of research institutions retain the potential to shift over time, if approached intentionally, to a position of shared leadership with communities [12].

### Foundational values of participatory praxis

A set of values focused on equity and engagement grounds participatory praxis (Table 1). Engaging stigmatized communities, including women, people living with illness or disability, lesbian, gay, bisexual, and transgender people, people of color, migrants, refugees, young people, economically disadvantaged people, and people who are institutionalized, requires researchers to un-learn practices that instill hierarchy and distance and a false researcher–subject dichotomy. With such training and reflexivity, researchers learn to make space for communities to determine the research questions and appropriate research methods.

At times, participatory praxis may seem unachievable, particularly for researchers who are not a member of the community that is being studied. Three points are essential to note. First, everyone exists at a particular intersection of privilege and oppression; acknowledging this fact is a first step in putting the values of accountability, non-othering, and dignity into practice. Second, a reflexive understanding of one’s own situatedness vis á vis different statuses of power and privilege is critical for effecting participatory praxis. Membership in a particular community is not a precursor to participatory praxis. In fact, even – and perhaps especially – researchers from the community must also navigate their privilege, for example, as related to institutional, economic, or educational status, as well as gender, race, dis/ability, and other statuses. Third, in order to uphold the values of equity, justice, and flipping power dynamics, participatory praxis must be viewed as a long-term mission. Un-learning practices that instill hierarchy and distance requires constant engagement and commitment to the the values listed in Table 1.

**Table 1:** Foundational values for participatory praxis in health-related stigma research

Equity	Research is designed and conducted with the goal of social egalitarianism that improves health and well-being for marginalized groups. Researchers and communities are co-equal investigators with different sets of skills and experiences to share with each other
Justice	Research is designed and conducted with the goal of creating social change that increases access to the rights and privileges of citizenship, including access to healthcare, recourse for discrimination, and voice in decision-making and policies for marginalized groups
Dignity	The inherent worth and value of all participants is recognized at every stage of the research
Participation	The people and communities that are the subjects of research should lead the research and be meaningfully engaged at all stages, including the right not to participate
Non-othering	People and communities who are the foci of research are seen as similar to the researchers, and approached from a common base of humanity, rather than seen as essentially different, exotic, incomprehensible, or ‘other’
Accountability	Communities and researchers hold themselves and each other accountable to their commitments, including to participatory praxis
Reflexivity	Researchers and communities engage in self-reflection to examine their own stigmatizing attitudes and biases and commit to rigorously seeking out and addressing their own prejudices and to refrain from acting on them
Transparency	The rules for decision-making are clear, collective, agreed in advance, and followed
Flipping power dynamics	Research is designed and conducted with the goal of creating social change that results in marginalized groups gaining greater control and self-determination over their lives and environments

Definitions created by the authors based on principles described in the literature [2, 4, 7, 9]

As participatory praxis strives to reveal power relations and expose biases, enriching the collective understanding of community strengths and needs, it contributes to an iterative cycle of learning and approaches that respond to changing needs [4]. This orientation calls for exchanging terms like ‘recruitment’ and ‘technology transfer’ for ‘partnership’ and ‘capacity exchange’ [13]. When these happen, participatory praxis facilitates a research process that is responsive to community-identified priorities, creates a sense of community ownership, foregrounds and addresses risks to communities from participating in research, and has the potential to undo stigma [14].

### Research led by the community, based on community-identified priorities

Participatory praxis ensures that, when research is conducted, it has been designed to meet community-identified priorities. In 2010, 2012, and 2014, MPact Global Action for Gay Men’s Health and Rights (formerly known as the Global Forum on Men Who Have Sex with Men and HIV) created and administered the Global Men’s Health and Rights study, a multilingual, international, online survey involving men who have sex with men [15–17]. MPact is a network of advocates and service providers working to ensure equitable access to health services for gay men and other men who have sex with men, while promoting their health and human rights worldwide. The survey gathered information about the barriers to and facilitators of HIV services. Findings documented strong associations between experienced homophobia, provider stigma, and compromised access to HIV services and revealed important associations between connections to gay community, access, and service utilization. Because the survey was designed by and for gay men and other men who have sex with men, advocates had the information that they needed to inform WHO guidelines on effective interventions for gay and bisexual men and field guidelines for implementing sensitized programs in low- and middle-income countries [18, 19]. The Global Men’s Health and Rights study helped advocates demonstrate the importance of community-led service delivery and why addressing stigma and discrimination is essential for optimizing service uptake.

### Community ownership of the research results

When participatory approaches are used, then communities own the process and the results, either fully or in partnership with formal researchers. Community ownership of research results leads to programming that can be responsive to evolving needs at multiple levels. For example, in 2014, young people living with HIV in Kenya and Uganda conducted surveys with other young people living with HIV, healthcare providers, and policy-makers to learn about access to sexual and reproductive health services and knowledge regarding the social, health, and prevention needs of young people living with HIV. The research demonstrated the critical role of providers in shaping sexual behaviors and fertility desires of young people living with HIV, yet found that provider-initiated information increased stigma and was not comprehensive. In 2015, Ugandan young people living with HIV used the survey findings to influence the Uganda Ministry of Health to include health promotion, access to integrated sexual and reproductive health, and rights and HIV services, as well as empowerment programs in the adolescent service and care package.

### Potential risks to community members foregrounded and addressed

Participatory engagement of stigmatized populations in research carries risk, including being jailed, attacked, or killed in repressive countries, and stigmatized or identified as a marginalized group member more generally [20–22]. Therefore, the perceived futility of participation or need for self-preservation may impact participation [22]. Alternately, marginalized populations may choose the risks of participation over those of doing nothing. As one researcher has noted regarding Indigenous young people in Guatemala, “[f]*or historically oppressed groups coming-of-age in high-risk settings, empowerment and endangerment are inevitably entwined*” [20].

Participatory praxis provides the context to support community voice and minimize risks. Establishing community advisory boards has facilitated youth participation and decreased barriers in HIV research [22]. Further, establishing partnerships with community organizations has enhanced recruitment of underserved communities in population health research projects [23]. In Lebanon, the participation of staff members from community-based NGOs serving men who have sex with men, injection drug users, commercial sex workers, and individuals in prison was critical in minimizing ‘harms’ from participation and enhancing benefits in a biobehavioral HIV survey [21]. While participatory praxis can appear to be an unaffordable luxury when conducting research in dangerous or repressive settings, in reality, stigmatized groups are in greater danger from research in which they are not meaningfully involved or in which they do not experience shared leadership. Community members understand their context and the risks they face and can train researchers in the best practices for engagement while protecting their confidentiality and safety.

### Undoing stigma

Engaging with communities as leaders, experts, and agents for change in addressing health stigma not only creates stronger and safer research studies and more relevant evidence, but can also directly affect the internal stigma that these communities face as a result of the stigma in societies. As one example, using a reflexive narrative methodology, Spieldenner et al. [24] explore the effects of participatory praxis on formal researchers and community members in three People Living with HIV Stigma Index implementations in the US. The author group, comprised of formal and community researchers who led the project, asked themselves a series of questions about the outcomes that they experienced through their work on the project. The text analysis identifies categories of change, including an increase in personal agency, as participants describe the transition from being viewed as a ‘consumer’, seen as a passive and patronizing identity, to being a researcher and a content matter expert, helping to collect data that is meaningful for their communities. They highlight the benefit for self-efficacy and self-esteem from working together on a common goal with other people similarly situated. They focus on moments when stigma among themselves and within the community emerged and used opportunities that participatory praxis provided to respond to that stigma. Finally, they note the high rates of economic fragility among people living with HIV who completed the survey. They expressed how important it was that the project did not expect people with HIV to work as volunteers but, instead, paid them for their work as project managers, trainers, interviewers, and for advisory work. Working in partnership with communities that are stigmatized creates awareness of what stigma looks like in concrete terms, and highlights to those outside the community how their actions and words may implicitly stigmatize.

Further evidence for the beneficial outcomes to communities of engaging in participatory research include greater overall health and mental health, bonding with others, and greater self-efficacy [12], while involvement in collaborative action for social justice, a critical component of participatory praxis, is linked to higher levels of political engagement over time [25] and an “*enhanced sense of self, belief in change, and empowerment*” [26].

### Supportive structural changes in funding, training, publication, promotion, and tenure processes

Beyond individual-level commitments to participatory praxis and shared leadership, structural changes will be necessary for a change in research culture that values equally the experiences of stigmatized communities. The training of researchers in the biomedical sciences largely focuses on a particular scientific approach, with its concomitant realism ontology, etic^1^ epistemology, and quantitative methods. Training of researchers in the health sciences as well as the social sciences also tends towards these paradigms. Yet, participatory research is based primarily on relativism, emic^2^ approaches, and mixed methods, with qualitative methods providing strong evidence. Even when facilitators working in participatory action research programs are trained in participatory methods, the potency of the scientific method interferes with their ability to engage most effectively to raise community voices [27]. For example, effective facilitation of participatory praxis entails disposing of the ‘expert’ cloak, as well as discarding the notion of one truth. Indeed, participatory praxis requires un-learning practices that instill hierarchy and distance. For participatory praxis to thrive, a fundamental shift in training of future researchers is critical for them to acquire competency in both positivistic and non-positivistic research traditions, and view them as equally robust, valid, and reliable. Guidance on how to begin this process can be found in the writings of community-engaged academics [28], but a paradigm shift is needed. Only recently, in 2016, did the Council on Education for Public Health include qualitative methods as a required foundational competency for masters and doctor of public health students in accredited public health programs and schools [29].

The positivistic approach has pervaded what are considered to be indicators of impact in research and what is, therefore, publishable. Cook and Roche, in an editorial to the recent special issue of *Educational Action Research*, focused on The Conceptualisation and Articulation of Impact: Hopes, Expectations and Challenges for the Participatory Paradigm, suggesting that “[f]*or participatory researchers and their partners (community members/practitioners/decision-makers), understandings of impact seldom map neatly onto conventional indicators or simplistic metrics. Research that has participatory practices at its centre is likely to have different types of impact from research that starts from a position of distanced objectivity*” [30]. One way that universities and academic centers can incentivize and reward the use of participatory praxis is by including information about community engagement in publications and including community stakeholders in review processes.

Engaging in participatory praxis requires a period of trust-building and reflexivity, ahead of the joint work of identifying needs and assets. Researchers and practitioners engaging in this type of work are often disadvantaged by tenure and promotion guidelines that are built on quicker quantitative positivistic approaches to research productivity. Further, these characteristics of participatory praxis are often also at odds with funding cycles and grant requirements. For real movement toward participatory praxis, funders will need to prioritize participatory praxis when funding health-related stigma research, perhaps through special requests for proposals. Health-related stigma research can reduce the health inequities faced by stigmatized groups if funders, institutions, and academic peer-reviewed journals require and reward participatory research and practice.

### Towards a research agenda for participatory praxis in health-related stigma research

An examination of current practices, gaps, and opportunities could enhance understanding of the state of participatory praxis in health-related stigma research and increase the use of such approaches or methods. A research agenda for participatory praxis in health-related stigma research is suggested in Box 1.

## Conclusion

Participatory praxis is an ethical imperative when conducting health-related stigma research. Yet, for community-engaged practice to become more than an ethical aspiration, and for researchers to be supported and encouraged to adopt these approaches, structural changes in the funding, training, promotion, publishing, and tenure processes will be necessary. A variety of resources are available to researchers committed to re-balancing the scales of equity and justice, and enhancing dignity for persons and communities who are stigmatized (Box 2).

Participatory praxis has the potential to balance historical injustice and enhance equity while achieving better health outcomes. However, health-related stigma research is frequently conducted without meaningful inclusion of those who are stigmatized in the research process. Such research risks reinforcing the prejudices that frame stigmatized people as less than full members of their communities or as problems to be solved (by others) rather than experts in their own experience and leaders in their own emancipation. Further, non-participatory research into health-related stigma may utilize what are often scarce resources for research on study designs that lack internal validity and are irrelevant for meeting community needs. By contrast, participatory praxis in health-related stigma research enriches the understanding of community strengths as well as health needs and priorities, and helps to balance the scales of equity. This research is designed to ensure that community priorities, rather than those of individual researchers, are centered in health-stigma research, that communities own the research results that they can use to advocate for better treatment, that proper attention and mitigation are provided for the potential risks that community members might face as a result of their participation in research, and that the act of engaging in research leads to undoing, rather than perpetuating, stigma.

**Box 1** A research agenda for participatory praxis in health-related stigma research A systematic review of existing health-related stigma research studies to document when, where, and how communities were engaged, with particular attention to documenting how participatory praxis is employed across different health conditions and stigma types.

Process evaluations that highlight the ways in which the values listed in Table 1 have been enacted for enhanced participatory praxis, and how they have affected the undoing of stigma and movement towards the goal of social change.

Research to identify the impact of using participatory praxis, considering innovative and non-traditional indicators.

Exploratory studies to identify differential values that community members and researchers bring to such processes, factors that facilitate more meaningful community engagement in research, mechanisms through which community participation in research influences internalized stigma and enacted stigma, links between participatory praxis and increased health equity, and secondary drawbacks and gains from the uses of participatory praxis in health-related stigma research.

Multi-method explorations and modeling to examine how structural changes could have a high impact for increasing participatory praxis, such as through shifts in funding priorities to favor the adoption of participatory methods in research projects studying health-related stigma, the use of participatory approaches and their timetables, and changes in university hiring and tenure processes.

**Box 2** Participatory praxis resources Participatory Praxis Resources for Health Research: A Starting Point

Minkler M, Wallerstein N (Editors). Community-based Participatory Research for Health. San Francisco, CA: John Wiley & Sons, Inc.; 2017.

Israel BA, Eng E, Shultz AJ, Parker EA (Editors). Methods for Community-based Participatory Research for Health. San Francisco, CA: Jossey-Bass, Inc.; 2012.

Principles of Community Engagement, Second Edition. The Clinical and Translational Science Awards Consortium, Community Engagement Key Function Committee, Task Force on the Principles of Community Engagement. 2011. Bethesda, MD: US National Institutes of Health. https://www.atsdr.cdc.gov/communityengagement/pdf/PCE_Report_508_FINAL.pdf. Accessed 17 Jan 2019.

CARE: Community Alliance for Research and Engagement. Principles and Guidelines for Community–University Research Partnerships*.* New Haven, CT: Yale University; 2009.

International HIV/AIDS Alliance, Academy for Educational Development, and International Center for Research on Women. Understanding and Challenging HIV-related Stigma and Discrimination: A Toolkit for Action. 2007. https://www.icrw.org/publications/understanding-and-challenging-hiv-stigma-toolkit-for-action/. Accessed 17 Jan 2019.

Wallerstein NB, Duran B. Using community-based participatory research to address health disparities. Health Promotion Practice. 2006;7(3):312–23. doi:10.1177/1524839906289376.

## Footnotes

1 Etic approaches focus on meanings that come *from outside* the community which is the subject of a study. Etic epistemologies are developed by communities of scientists and researchers, using conceptual categories and hypotheses that are agreed to be important and are viewed as universal, or as undergoing hypothesis testing for potential universality.

2 Emic approaches focus on meaning as understood *within* a particular group of people. Emic epistemologies can be identified only by close listening to and engagement with the community that is the subject of a study.
